# Proving of Convallaria Majalis

**Published:** 1885-06

**Authors:** Irvin J. Lane

**Affiliations:** Sing Sing, N. Y.


### PROVING OF CONVALLARIA MAJALIS.

Irvin J. Lane, M. D., Sing Sing, N. Y.

Having proved Convallaria majalis tincture some time ago, and finding that it was a very important remedy, I thought it should be proven in a dilution, so July 1st, 1884, I commenced taking Convallaria majalis, third centesimal dilution, prepared from the flowers and upper part of the stems of the "Lily of the Valley," by chopping and pounding them to a pulp, after which I added an equal part, by weight, of alcohol, then stood it in a cool place, shaking it occasionally for eight days, then filtering it, and preparing the dilutions, as directed under Class II of the Homœopathic Pharmacopœia.

At the time I commenced the proving I was in good health, with the exceptions of a slight cold and a collection of phlegm in the throat mornings, which disappeared after breakfast. The above symptoms, or any other that I had had for some time previous to the commencement of this proving I was very particular in guarding against recording.

Age twenty-three; eyes blue, hair brown, form stout, features full, chest large and full, habits good, bowels regular.

I saturated disks, so that each disk would absorb one drop, and commenced July 1st, 4 P. M., taking one disk at a time every two hours.

July 7th.—Each day, since I commenced taking Convallaria, I have had two stools a day. This afternoon, between 3 and and 5 o'clock, I have felt very sleepy and weak. A small aneurism, about the size of a millet seed, formed on my left index-finger, which, after picking it with a pin, bled quite profusely and was difficult to stop; the slight bleeding caused a faint feeling and I turned pale. During the day whenever I would feel a little cold I would have a chill. During the chill I would be very nervous, so that my hands would tremble.

July 8th.—Feel very sleepy between 3 and 5 P. M.

July 10th.—Smarting of the right eye at about 12 M., which lasted only a few minutes; grieve very easily; feel sleepy at about 6 P. M., while sitting; food eaten at a restaurant did not look or taste fit to eat, as it did not seem clean.

July 11th.—Forenoon; eyes felt dry, and a sensation, as if the inner parts of the lids were rough, which I felt when first awaking, and lasted until about 10 A. M.; food did not taste as good as common; appetite diminished; very sleepy after dinner.

July 13th.—Was quite nervous; my hand would tremble when trying to give a spoonful of medicine, so that I could not take a full spoon; grating of the teeth mornings as soon as I began to awake.

July 15th.—Eructation after eating cucumbers, tasting of the cucumbers; pain in left part of umbilical region, after walking a short distance; food does not seem as if it was clean and fit to eat; thirsty, drinking a great deal of water, especially in the evening; feel sleepy and lazy.

July 18th.—While eating dinner I had a colic-like pain in the umbilical region, which extended to the right lumbar region; two stools each day.

July 19th.—Colic in hypogastric region this morning, before I got up, which lasted until my bowels moved.

July 21st, 10 P. M.—After urinating there was an aching in region of bladder, as if the bladder was over-distended, with aching and lame feeling in the back after lying down.

July 22d, A. M.—Examination of urine passed since yesterday afternoon. Color light brown, odor strong, smelling like fresh fish; specific gravity 10.30, acidity normal, slight mucous sediment after standing; albumen none, sugar normal, amount about normal, but urinate more frequently than common.

July 24th, 6.30 A. M.—The skin all over the body feels sore on awaking and after rising; pain in the lumbar region, as if the muscles were bruised.

7 A. M.—Headache, commencing in the forehead; thirsty, drinking large quantity at a time, and often. No appetite, eat only a small dish of blackberries.

8.30 A. M.—Shortness of breath while walking. Fever commenced about 8.15 A. M.; at 8.30 temperature was 104½, pulse 124; headache in the frontal region, of a dull, heavy ache, and severe dull aching in the lumbar region, as if the back had been pounded. I went to bed, and although I kept my eyes closed all the while I could not sleep, but would hear everything; legs ached during the fever, which lasted until about 11 A. M., when I commenced to sweat. During the fever I could not hear as well as usual; the ears seemed to be stopped; could feel the heart beat throughout the chest. Whenever I would move any part it would cause a chilly sensation all over the body; motion would aggravate the headache, but felt better after moving around and talking.

Skin felt rough while washing, after perspiring.

12.15 P. M.—Legs ache. Dull, heavy, pressive headache in the frontal region; had to step very light, as the least jar would aggravate the headache. Backache very severe, as if bruised or broken. Temperature 104, pulse 106. Collection of tough, clammy mucus in the mouth; appetite very poor.

1.30 P. M.—Temperature 102; backache and headache better; absence of thirst after the fever commenced.

2.20 P. M.—Aching of head, back, and legs still continues, but not as severe. Temperature 102, pulse 92. Urine dark colored.

3 P. M.—Dullness of intellect; can hardly think of the remedy I want to prescribe for a patient. Headache worse while walking up hill; aching in frontal region and nape of neck while walking, so that I had to step very light to prevent any jarring of the head; worse while walking up hill. Dyspnœa while walking up hill.

(Stopped taking Convallaria.)

4.30 P. M.—Coppery taste in the mouth; dullness of intellect; heavy, pressive aching in frontal region.

7 P. M.—Headache aggravated by stepping heavy or jarring the head, gradually passing away after supper.

10 P. M.—Urine very dark colored and strong-smelling; headache only when jarring or shaking the head.

July 25th.—Feel very well this morning except a frontal headache, which I would notice only when jarring the head. At times during the day there would be a pricking sensation on the forehead, as if touched with nettles, with no eruption, worse after getting very warm.

The headache lasted until 4.30 P. M., when I commenced to have a fever. As the fever increased, so did the headache; did not notice the headache but very little, except when I would move or jar the head, when it would be very severe; thirst during the first part of the fever, trembling of the hands and legs during the fever, aggravated by a draft of air; coppery taste in the mouth.

5 P. M.—Temperature 103$\frac{2}{5}$, pulse 106. Hot feeling in the thorax and hypochondria; weak, empty feeling in the pharynx, extending to the stomach; dyspnœa during the fever, with a desire to take a deep breath; back aches a very little.

6 P. M.—Tired out feeling after walking, with aching in the legs and back and aggravation of the headache; urine scanty, dark colored, and smells like cows' urine.

7 P. M.—Appetite poor; sleepy during the fever; by keeping quiet it relieves the headache; dyspnœa. Cold chills would commence in the back and run down the spinal column from a draft of air or by taking a drink of cold water; water felt cold all the way down the œsophagus; sensation as of bubbles of air passing through water from symphysis pubis to the right side of hypogastric region; dull, colic-like pain across the umbilical region.

8 P. M.—Pulse 100. Gurgling of water in the umbilical region; legs ache some yet, mostly the right, and below the knee; headache more in the right temple.

11 P. M.—Headache aggravated by hawking to clear the throat, which caused very severe pains.

July 26th.—Was chilly all night; awoke about 2 A. M., was awake for a few minutes, then fell asleep again; awoke again at 4 A. M. and felt quite chilly, then went asleep again; before getting up, I had a dull, pressive headache in the frontal region and temples, more on the right side. Chills would run along the back on moving any part of the body; I laid for about an hour before arising, but could not get asleep; did not want to get up, on account of the headache, which was aggravated by the least motion; nauseous feeling in the stomach.

7.15 A. M.—As soon as I got out of bed I commenced shaking with a chill all over the body; ends of fingers were pale, with the rest of the hand mottled purple and white; lips purple and sore, and covered with hydroa; headache quite severe, aggravated by motion or stooping, so that I would step as light as possible to prevent jarring the head; hair was sensitive to touch; chill was ameliorated by the heat of the stove or covering up warm; water tastes bitter; no appetite for breakfast; drank a glass of water and part of a cup of coffee; tongue felt as if scalded when drinking the warm coffee; thick, clammy saliva in the mouth; headache very severe, of a dull, pressive character, with backache and aching of the legs; nausea during the chill, which lasted about one hour; headache, backache in the lumbar region, and aching of the legs continued during the chill; could not get asleep, although I lay very quiet, with eyes closed; time passed very slowly; did not want to get up, on account of motion aggravating the headache and other symptoms.

9.30 A. M.—Desire for stool without effect; soreness of anus on straining, as if the anus was raw; urine scanty and very dark colored. While walking on the street I had to walk very slow, as I felt very weak, and would step very light to prevent jarring the head. Eyes felt heavy; I wanted to keep the lids closed all the while, but when I would close the eyes while walking I would stagger or walk sideways; dullness of intellect.

10.30 A. M.—After walking about a mile I was tired out, so that I had to lie down and rest. Felt chilly from draft of air or when lying on a cold sofa; headache, backache, and legs ache; tried to get asleep but could not; time passed very slowly.

12.30. P. M.—Some fever since the chill, but did not feel very warm. On getting up at about 12.15 P. M. I staggered and felt weak; appetite poor; desire for lemonade or something acid; after dinner I began to feel better.

2 P. M.—Temperature $102\frac{3}{5}$; tongue coated yellow at the base; the tip and sides are dotted red; tip of tongue feels sore, as if it had been scalded.

3 P. M.—Temperature 102, pulse 97, and quite weak; headache has nearly all passed away, but there is a dullness of the head; aching of the back in the lumbar region; aching of right big toe; urine lighter colored and more profuse.

5 P. M.—Cramps in calf of left leg while walking; head aches only a little, but feel weak, and back aches; dullness of head; hair very sensitive to touch; coppery taste in the mouth; do not feel like talking.

6.30 P. M.—Appetite for supper very good; left side of lips near the corner of the mouth is very sore, and covered with small hydroa; soreness of inner edge of the left nostril, caused by little blisters like hydroa, which feel as if the flesh had been torn, leaving a raw surface.

10 P. M.—Desire for stool, with very small fæces after prolonged tenesmus, as if there was inactivity of the rectum; tenesmus after stool.

11 P. M.—Have felt very well all the evening, with the exception of backache and weakness.

July 27th.—Slept well last night and feel very well this morning, except a slight weakness and fever blisters on the lips, which feel as if they were raw; tip of tongue sensitive and dotted with fine red points; appetite good; trembling of the hands.

1 P. M.—While I was eating dinner and sitting in a draft (the air was chilly and damp), cold chills commenced between the shoulders and ran down the back, extending to the legs. Ends of fingers turned pale, and felt very cold; the chill was ameliorated by wrapping a heavy blanket around my shoulders and sitting in a room where there was no draft. Appetite for dinner not very good—desire for something acid.

2 P. M.—Chill is not so severe, but I notice it more the harder it rains; whenever touching any part of the body with anything cold, it will cause a chill to commence between the scapula and pass down through the legs. Pressing, aching sensation at the anus, with desire for stool, but nothing but flatus passes; raw feeling in posterior part of the pharynx when inspiring, ameliorated for a moment by hacking cough; feel dull and sleepy, with desire to go to bed.

3.45 P. M.—The chill continued for about twenty minutes after lying down, then gradually passed away, followed at 2.20 P. M. by a fever, commencing in the back between the scapula by a hot spot covering a place about four inches in diameter, then extending to the right side of the head, then all over the head and down the back; the heat is not very marked in the legs, which may be due to not having them covered, as the upper part of the body was; the fever passed away in reverse order from which it came; sensation as if the bladder was over-distended, with passage of only a small quantity of urine; stool followed by tenesmus; not much thirst during the chill or fever.

4 P. M.—Slight perspiration.

5 P. M.—Heavy, pressive headache, commencing after walking a few blocks, aggravated by motion; abdomen sensitive to pressure, clothes feel tight.

6 P. M.—Appetite for supper very good; headache gradually passed away after lying down; inner edge of left nostril covered with small hydroa, which are very sore and smart; lips covered with small hydroa, which are very sore, left side worse.

7.30 P. M.—Headache on going up-stairs.

10 P. M.—Headache on going up-stairs, not while walking around or going down-stairs; pain in the abdomen, across the hypochondriac region during deep inspiration or gaping.

July 28th.—Epistaxis about 2 A. M., awaking me from sleep.

6.30 A. M.—Felt weak after getting up, so that I had to walk very slowly while out walking; appetite not very good.

8 A. M.—Chill commenced in the back and soon extended all over the body; wanted to wrap up very warm, which would ameliorate the chill; I sat with my back toward the stove, as my back seemed colder than any other part of the body; the heat of the stove would ameliorate the chill while I sat there; while walking on the street, the chill was so severe that I could not keep my teeth from chattering; the ends of the fingers were white, and the first joint of the index-finger of the right hand was numb, and remained so during the chill; the chill lasted about one hour, when it gradually passed away as the fever commenced to come on; continued covered up during the fever, which lasted until 11.30 A. M., when it gradually passed away, and was followed by slight perspiration; not much headache during the chill or fever.

12.20 P. M.—Appetite for dinner very good. After dinner I felt very well, except being somewhat weak, and a feeling of weakness in region of the stomach.

6 P. M.—Feel very well; appetite for supper good.

8 to 10 P. M.—After leaning over for some time my back ached, with a very lame and weak feeling in the dorsal region. Hydroa on the lips and nose better. Pain in the region of the gall bladder on taking a deep breath or raising the arms.

July 29th, 5.45 A. M. (raining).—I got up and my head commenced aching severely, as if I had slept too sound, so I lay down again until 6.40 A. M., when the headache was some better. I moved around very slowly, so as not to aggravate the headache, which gradually passed away about 8.15 A. M. Appetite poor. Examination of urine, sample taken from that passed during the last twenty-four hours—color dark, odor strong and offensive, specific gravity, 10.24, slightly acid, sugar normal, no albumen.

During the remainder of the day I felt very well with the exceptions of a weak feeling in the abdomen and a weak and dull feeling of the head. The hydroa on the lips are very sore and feel as if the lips were raw; hydroa in the nose are healing up.

July 30th.—Did not want to get up. Headache through the fronto-temporal regions, worse on the right side, of a dull, pressive character, which gradually passed away.

Evening.—During the day since the headache stopped I have felt very well; the hydroa are gradually healing.

July 31st.—Headache on rising as if I had slept too sound; aggravated by motion. No desire to get up in the morning.

The trembling of the hands, which commenced during the first part of the proving, continued for five or six weeks after I stopped taking the medicine.

For convenience of reference or study, I will arrange the symptoms of the above proving according to the part affected. The symptoms in italics were more marked than the others; they either occurred oftener, were more severe, or lasted longer.

MIND.—Dullness of intellect; grieve very easily; aversion to talking.

HEAD.—Dull, heavy, pressive headache in the frontal region; headache aggravated by walking up hill, going up-stairs, ascending, jarring the head, motion, leaning over, or hawking to clear the throat; aching of frontal region and nape of the neck while walking up hill, so that I would have to step very light to prevent jarring the head; severe pain in the head when hawking to clear the throat; headache worse on the right side; headache on getting up in the morning, as if I had slept too sound. Dull, pressive headache through the fronto-temporal region, worse on right side; hair sensitive to touch.

EYES.—Smarting of the right eye at about 12 M., which lasted only a few minutes; when first awaking in the morning the eyes felt dry, with a sensation as if the inner part of the lid was rough, which lasted until 10 A. M.; eyes felt heavy, wanted to keep them closed.

EARS.—Dullness of hearing during fever.

NOSE.—*Hydroa in the nose; little blisters like fever blisters on inner side of left nostril, which are very sore, as if the flesh had been torn, leaving a raw surface;* epistaxis at 2 A. M., awaking me from sleep.

FACE.—Pricking sensation on the forehead, as if touched with nettles. *Lips covered with hydroa, which were very sore, as if the lips were raw; left side of lips and corner of the mouth were the worse.*

MOUTH.—*Grating of the teeth in the morning, as soon as he began to get awake*; collection of tough, clammy-like mucus in the mouth; coppery taste in the mouth; tongue coated yellow at the base; tip and sides dotted red; tip of tongue felt sore, as if it had been scalded; tongue felt as if scalded when drinking warm coffee.

THROAT.—Weak, empty feeling in the pharynx, extending to the stomach; raw feeling in the posterior part of the pharynx when inspiring, ameliorated for a moment by a hacking cough.

STOMACH.—Food eaten at a restaurant seemed unclean and unfit to eat; even food eaten at home seemed unclean, and did not look or taste good, especially meat stews; sensation as of weakness in the region of the stomach; food did not have its natural taste; appetite changeable; eructations after eating cucumbers, tasting of cucumbers; desire for lemonade or something acid; thirsty, especially in the evening. While drinking, water felt cold all the way down the œsophagus; water tastes bitter during fever; nausea in the morning during chill.

ABDOMEN.—Pain in left part of umbilical region after walking; colic-like pain in umbilical region, extending to right lumbar region, while eating dinner; colic-like pain in the hypogastric region before getting up in the morning, lasting until after stool; pain in the region of the gall bladder on taking a deep breath or raising the arms; pain in the abdomen across the hypochondriac region on taking a deep breath or gaping; dull, colic-like pain across the umbilicus; abdomen sensitive to pressure; clothes feel too tight; weak feeling in the abdomen; hot feeling in the hypochondriac region and thorax; sensation as of bubbles of air passing through water, from the symphysis pubis to the right side of the hypogastric region; gurgling as of water in the umbilical region.

STOOL AND ANUS.—Desire for stool without effect, or with very small fæces after prolonged tenesmus, as if there was inactivity of the rectum; tenesmus after stool; pressing, aching sensation at the anus, with desire for stool, but nothing but flatus passes; soreness of the anus on straining to stool, as if the anus were raw.

URINARY ORGANS.—Aching in the region of the bladder after urinating, as if the bladder were over-distended, with an aching, lame feeling in the back after lying down; sensation as if the bladder was over-distended with passage of only a small quantity of urine; micturition frequent; urine smelling strong, offensive, like fresh fish, or like cow's urine; color dark, or light brown.

RESPIRATORY ORGANS.—Dyspnœa while walking; dyspnœa while walking up hill; hot feeling in the thorax and hypochondriac region; dyspnœa during fever.

HEART AND PULSE.—Feeling of the heart beat throughout the chest.

NECK AND BACK.—Pain in the lumbar region, as if the muscles had been bruised; dull, aching pain in the lumbar region, as if the back had been pounded; backache, with lame and weak feeling in the dorsal region, after leaning over for some time.

UPPER LIMBS.—Numbness of the first joint of the index-finger of the right hand during chill.

LOWER LIMBS.—Aching of the legs, worse below the knees; aching of right big toe; cramps in calf of left leg while walking.

GENERALITIES.—Formation of a small aneurism about the size of a millet seed, which bled profusely when opened and was difficult to stop; faint and pale from slight loss of blood; *trembling of the hands;* tired-out feeling when walking, with aching of the legs and back; weakness; eyes felt heavy, wanted to keep them closed all the while, but when closing them while walking I would stagger or walk sideways.

SKIN.—Sensation in the morning as of soreness of the skin all over the body; skin felt rough while washing, after perspiring; pricking sensation on the forehead during the day, as if touched by nettles, aggravated by warmth, no eruption.

SLEEP.—Time passed away very slowly when trying to get asleep; sleepy and weak between 3 and 5 P. M.; sleepy after dinner; no desire to get up in the morning.

FEVER.—*Chill whenever I would feel the least cold.* Nervous, with trembling of the hands during chill. Chilly all over body on moving; *chills would commence in the back and run down the*

*spine from a draft of air or by taking a drink of cold water.* Chills would creep along the back on moving any part of the body; chill was ameliorated by the heat of the stove or by covering up warm. Nausea in the morning during chill; weakness during the chill; chills commence in the back, between the shoulders, and run down the back and legs; chill commencing between the scapula and passes down the back and legs whenever any part of the body is touched by anything cold. During the chill the ends of the fingers were pale, while the rest of the hand was mottled purple and white; ends of fingers pale and very cold; numbness of the first joint of the index-finger of the right hand during chill; chattering of teeth during the chill, while walking on the street; chill worse the harder it rains. *Headache during chill; paroxysm of chill, fever, and sweat in the forenoon of one day and afternoon of the next day.*

*The fever is the principal part in intermittent fever, as the chill is light, and sweating stage very light, or entirely wanting.* Thirst and headache during chill or preceding the fever; sleepy during the fever, but could not sleep, as I would hear every little noise. *Headache and aching of the back and legs during the fever; fever preceded by no marked chill, and followed by slight perspiration.* During the fever the ears seemed stopped up, so as to cause a dullness of hearing. Headache increased as the fever increased. Dyspnœa during the fever, causing me to take a deep breath quite often; desire to be covered during the fever; easily exhausted while walking; water tasted bitter during fever. Fever commencing in the back, between the scapula, by a hot spot about four inches in diameter, then extending to the right side of the head, then all over the head and down the back and all over the body. The fever passed off in reverse order from which it came. *Headache, backache, and aching of the limbs continued during and after the chill, fever, and sweat.* Sweating stage may be entirely wanting.

AMELIORATION.—By warmth.

AGGRAVATION.—During rain; by motion; by cold air.

---

WOMAN'S HOMŒOPATHIC ASSOCIATION OF PENNSYLVANIA.—Organized under the above title, some philanthropic ladies interested in Homœopathy have established an hospital and a training school for nurses in this city. With untiring energy they have collected funds and are erecting a fine hospital in the northern section of the city. It is the desire of the Managers to have true Homœopathy taught and practiced in this hospital; in this endeavor all will wish them the best success.

There are two courses of lectures yearly, Fall and Spring, delivered at the hospital by prominent homœopathic physicians.

